# Idarucizumab in High-risk Thoracic Surgery

**Published:** 2018-05-01

**Authors:** R. López-Vilella, J. Sanz-Sánchez, I. Sánchez-Lázaro, E. Marques-Sule, J. Rueda-Soriano, L. Almenar-Bonet

**Affiliations:** 1 Heart Failure and Transplantation Unit, Cardiology Department, Hospital Universitario y Politécnico La Fe, Valencia, Spain; 2 Physiotherapy Department, University of Valencia, Valencia, Spain; 3 Adult Congenital Heart Disease Unit, Cardiology Department, Hospital Universitario y Politécnico La Fe, Valencia, Spain

**Keywords:** Heart-lung transplantation, Anticoagulants, Idarucizumab

## Abstract

Direct oral anticoagulants have suggested a favorable profile compared with vitamin K antagonists. However, the lack of treatment to reverse the effect of direct oral anticoagulants has limited its use in some patients who require rapid reversal of anticoagulation, as those included in the transplant waiting list. Idarucizumab is a recently approved drug to reverse the anticoagulant effect of dabigatran. However, the clinical experience when using this drug is scarce. Herein, we present a clinical case on anticoagulation reversal with idarucizumab to perform heart and lung transplantation in a patient with Eisenmenger syndrome.

## INTRODUCTION

Dabigatran is a direct thrombin inhibitor with favorable effectiveness and safety profile when compared with vitamin K antagonists (VKAs). Before idarucizumab’s approval, VKAs were the main therapy for patients waiting for transplantation who required long-term oral anticoagulation, as antidotes for direct acting oral anticoagulants (DOACs) were not available. Idarucizumab is the first targeted reversal agent specific for dabigatran. It is approved for emergency surgery/urgent procedures or for life-threatening or uncontrolled bleeding in patients taking dabigatran. We present a patient with a very high risk of hemorrhage receiving dabigatran who required heart and lung transplantation.

## CASE PRESENTATION

Our patient was a 33-year-old man with congenitally corrected transposition of the great arteries and ventricular septal defect (VSD). Cardiac MRI showed a dilated right ventricle with an indexed end-diastolic volume of 165 mL/m^2^ on the left side, and moderate systolic dysfunction, having an ejection fraction (EF) of 36%. In addition, morphologically, left subpulmonary ventricle was observed on the right side with moderate depression (EF of 37%). The VSD was subpulmonary and presented a pulmonary/systemic flow rate (Qp/Qs) of 1.8. Right cardiac catheterization showed severe pulmonary hypertension (mean pulmonary arterial pressure of 92 mm Hg, systolic pulmonary arterial pressure of 109 mm Hg, and diastolic pulmonary arterial pressure of 70 mm Hg). Considering these findings, an implantable cardioverter defibrillator (ICD) was provided as the primary prevention for the sudden death, and treatment with bosentan was started. One year later the patient’s functional condition worsened; the maximal oxygen uptake in the exercise testing was 41% of the theoretical. Bosentan was replaced by sildenafil, but due to poor tolerance to sildenafil, Ambrisentan was prescribed.

Five months later, the patient was hospitalized for atrial flutter. During electrophysiological study, atrial fibrillation (AF) was induced, and cavotricuspid isthmus ablation and isolation of pulmonary veins were performed. The patient was then discharged in sinus rhythm and anticoagulated with 110 mg of dabigatran every 12 hours. Nevertheless, his clinical status progressively worsened in the following months, with limiting dyspnea and symptoms of low cardiac output. An upgrade from ICD to cardiac resynchronization therapy was performed, since the patient presented a high percentage of ventricular stimulation and reduced ejection fraction. A risk assessment study for heart and lung transplantation (HLT) was carried out. The echocardiogram showed severe biventricular dysfunction with severe tricuspid regurgitation. Right cardiac catheterization confirmed pulmonary hypertension; he had a mean pulmonary arterial pressure of 90 mm Hg, systolic pulmonary arterial pressure of 104 mm Hg, diastolic pulmonary arterial pressure of 72 mm Hg, pulmonary capillary wedge pressure of 36 mm Hg, pulmonary vascular resistance of 9 Wood units, and cardiac output of 2.92 L/min. No absolute contraindications for HLT were detected. The patient was included in the waiting list for transplantation and was discharged with anticoagulation therapy (dabigatran), after checking idarucizumab was available.

In October 2016, an optimal donor was found. The patient was admitted to the hospital 7 hours after the last dose of dabigatran (110 mg). Two ampoules of intravenous idarucizumab 2.5 mg were given over 5 minutes each, with an interval of 15 minutes between the first and second doses. Since the antidote is a non-competitive inhibitor, the onset of inhibition of the anticoagulant action of dabigatran is practically instantaneous. Therefore, the drug was administered once it was confirmed the donor was suitable and the transplant could be performed. Dabigatran mainly prolongs the activated partial thromboplastin time (APTT) and, to a lesser extent, the prothrombin time (PT). However, idarucizumab routine monitoring is not necessary due to its stable and predictable pharmacokinetics [1]. The patient’s APTT and PT before CPT was 41 and 14.3 seconds, respectively. HLT was performed without hemorrhagic, intra-operative or post-operative complications. 

## DISCUSSION

HLT is a surgical treatment for patients with end-stage cardiac or pulmonary diseases when they coexist with severe pulmonary or cardiac involvement, respectively [2]. Hospital mortality is high, close to 50% in some series [3], and it is related, to a great extent, to the high incidence of hemorrhagic complications and reinterventions for bleeding. The available data reveal the difficulty and relevance of hemostasis in patients undergoing HLT. These are terminal patients who require prolonged extracorporeal circulation, with hypothermia, and who undergo transplantation in a limited situation regarding the function of their vital organs, with early or established renal and hepatic failure.

In our patient, the etiological diagnosis was Eisenmenger syndrome. These patients have a pro-hemorrhagic condition because they frequently have thrombocytopenia [4], increased platelet volume and a significant reduction of platelet aggregation. Circulating platelets in peripheral blood are formed by fragmentation of the megakaryocytes produced in the bone marrow. This fragmentation occurs in the pulmonary vascular bed, but in patients with a right-to-left shunt, part of the venous blood flow passes directly to the arterial circulation without passing through the lungs. These patients present with increased number of megakaryocytes in the peripheral blood and decreased number of platelets, inversely proportional to the hematocrit and to the size of the shunt. Moreover, not only is there a decrease in the number of platelets, but their function might also be altered. A deficiency of vitamin K-dependent coagulation factors (II, VII, IX, X) and factor V have also been detected, as well as increased fibrinolytic activity and a deficiency of von Willebrand factor. Therefore, hemorrhagic complications in Eisenmenger syndrome are frequent [5, 6], as well as thrombotic complications. Despite this situation, it is common that patients with congenital heart disease have an indication for anticoagulation, for instance when presenting with arrhythmias, especially AF or atrial flutter, as in our patient. In this regard, direct-acting anticoagulants have proven to be at least as effective as anti-vitamin K (AVK) drugs for the prevention of cardioembolic stroke in patients with non-valvular AF. Moreover, direct-acting anticoagulants have proven to be safer than AVK drugs, since they present less frequently significant hemorrhages, especially intracranial hemorrhages [7]. Nevertheless, the absence of antidotes with a fast, effective and safe mechanism of action for reversal of anticoagulation has been one of the potential drawbacks for a greater use. Until now, when including a patient in a heart or heart-lung transplant waiting list, it was necessary to change the usual treatment for acenocoumarol, in order to effectively reverse anticoagulation prior to transplantation. Our patient was being treated with dabigatran, a direct thrombin inhibitor used to prevent embolic events in patients with non-valvular AF [7]. It was decided to maintain treatment with the direct-acting anticoagulant when including the patient in the waiting list because idarucizumab was available. Idarucizumab is a specific reversal agent for dabigatran therapy, and has recently been approved by the US Food and Drug Administration and the European Medicines Agency [8-12]. It is a humanized monoclonal antibody fragment that specifically binds to dabigatran and its metabolites with high affinity, with 300-fold stronger binding affinity to thrombin than dabigatran. The idarucizumab-dabigatran complex, with no intrinsic procoagulant activity, is characterized by a fast association constant and an extremely slow dissociation constant, resulting in a very stable complex.

The results of the phase III RE-VERSE AD study showed that idarucizumab neutralizes the effect of dabigatran in a matter of minutes in patients with severe bleeding, or in those who are undergoing emergency procedures [13]. Due to the mechanism of action of the antidote (non-competitive inhibitor), the onset of inhibition of the anticoagulant action of dabigatran is practically instantaneous. It should be noted that the specificity of the reversing agent is exclusively by dabigatran, so effects are only observed in thrombin bounded to the reversible agent. Therefore, there are no side effects derived from the actions of thrombin on platelets, fibrinogen or on the own ability to generate thrombin. Therefore, there are no procoagulant or anticoagulant effects intrinsic to idarucizumab. Routine monitoring of idarucizumab administration is not necessary, because of its stable and predictable pharmacokinetics [1]. HLT was performed without complications, since no hemorrhagic, intra-operative or post-operative complications were reported. 

The availability of a specific reversal agent is an important factor to consider when choosing an anticoagulant in clinical practice. There are some clinical cases or short series of published cases about the use of idarucizumab. Ours was the first case of reversal of anticoagulation with idarucizumab for an elective HLT. Firstly, a rapid reversal of the anticoagulant effect after administration is demonstrated. Secondly, efficacy and ease of use are illustrated, as well as the role in patient safety when a rapid reversal of the anticoagulant effect of dabigatran is required.

## CONFLICTS OF INTEREST:

None declared.

## FINANCIAL SUPPORT:

None.
